# The correlation between different ultrasound planes and computed tomography measures of abdominal aortic aneurysms

**DOI:** 10.1002/ajum.12319

**Published:** 2022-10-12

**Authors:** Brigid G Hill, Rossi Holloway, Joyce Lim, Kari Clifford, Sarah Lesche, James Letts, Jolanda Krysa

**Affiliations:** ^1^ Department of Surgical Sciences, Otago Medical School University of Otago Dunedin New Zealand; ^2^ Department of Surgery Te Whatu Ora Health New Zealand, Southern Dunedin New Zealand; ^3^ Department of Radiology Te Whatu Ora Health New Zealand, Southern Dunedin New Zealand

**Keywords:** abdominal aortic aneurysm, coronal plane, decubitus, measurements, ultrasound window

## Abstract

**Introduction:**

Ultrasound measurements of the aorta are typically taken in the axial plane, with the transducer perpendicular to the aorta, and diameter measurements are obtained by placing the callipers from the anterior to the posterior wall and the transverse right to the left side of the aorta. While the ‘conventional’ anteroposterior walls in both sagittal and transverse plains may be suitable for aneurysms with less complicated geometry, there is controversy regarding the suitability of this approach for complicated, particularly tortuous aneurysms, as they may offer a more challenging situation. Previous work undertaken within our research group found that when training inexperienced users of ultrasound, they demonstrated more optimal calliper placement to the abdominal aorta when approached from a decubitus window to obtain a coronal image compared to the traditional ultrasound approach.

**Purpose:**

To observe the level of agreement in real‐world reporting between computed tomography (CT) and ultrasound measurements in three standard planes; transverse AP, sagittal AP and coronal (left to right) infra‐renal abdominal aortic aneurysm (AAA) diameter.

**Methodology:**

This is a retrospective review of the Otago Vascular Diagnostics database for AAA, where ultrasound and CT diameter data, available within 90 days of each other, were compared. In addition to patient demographics, the infrarenal aorta ultrasound diameter measurements in transverse AP and sagittal AP, along with a coronal decubitus image of the aorta was collected. No transverse measurement was performed from the left to the right of the aorta.

**Results:**

Three hundred twenty‐five participants (238 males, mean age 76.4 ± 7.5) were included. Mean ultrasound outer to the outer wall, transverse AP and sagittal AP diameters were 48.7 ± 10.5 mm and 48.9 ± 9.9 mm, respectively. The coronal diameter measurement of the aorta from left to right was 53.9 ± 12.8 mm in the left decubitus window. The mean ultrasound max was 54.3 ± 12.6 mm. The mean CT diameter measurement was 55.6 ± 12.7 mm. Correlation between the CT max and ultrasound max was *r* ^2^ = 0.90, and CT with the coronal measurement *r* ^2^ = 0.90, CT and AP transverse was r2=0.80, and CT with AP sagittal measurement was *r* ^2^ = 0.77.

**Conclusion:**

The decubitus ultrasound window of the abdominal aorta, with measurement of the coronal plane, is highly correlated and in agreement with CT scanning. This window may offer an alternative approach to measuring the infrarenal abdominal aortic aneurysm and should be considered when performing surveillance of all infra‐renal AAA.

## Introduction

Abdominal aortic aneurysm (AAA) has a prevalence in the general population of approximately 4.8%.^1^ Infrarenal AAA is the most prevalent type, with the risk of rupture related to the absolute diameter. AAA occurs secondary to weakening of the aortic arterial wall, which increases with age, progressing to further dilatation and decreased wall thickness, resulting in increased wall tension and potential rupture and life‐threatening haemorrhage. Rupture is often the first clinical presentation of an AAA and has a significantly high mortality rate of 81%.^2^

The detection of AAA by physical examination of the abdomen has been shown to have a sensitivity of 68% and specificity of 75%. The diagnostic accuracy is significantly reduced with obesity, abdominal distension and smaller aneurysm sizes.^3^ Ultrasound is the standard method for screening AAA and surveillance of the aortic measurement to determine growth rates over time.^4^, ^5^ Ultrasound screening and surveillance have significantly reduced AAA‐related mortality. Low cost, ease of accessibility, non‐invasiveness and safety make ultrasound an attractive modality for screening and surveillance programmes.^6^ However, its diagnostic quality is user‐dependent. Confirming the size of the abdominal aorta is essential for both progression of growth and the growth rate.^7^, ^8^, ^9^

Ultrasound measurements of the aorta are typically taken in the transverse plane, with the transducer perpendicular to the aorta, and diameter measurements are obtained by placing the callipers from the anterior‐to‐posterior wall and the lateral‐to‐lateral wall of the aorta.^5^, ^6^, ^7^, ^8^, ^9^ The anterior‐to‐posterior measurement can also be retaken in a sagittal plane by moving the transducer parallel to the aortic wall. The placement of the aorta's posterior wall callipers in both the transverse and sagittal planes has been discussed for some time. There is an ongoing debate on which layer of the aortic wall to place the callipers, whether the leading‐to‐leading, inner‐to‐inner or outer‐to‐outer edge should be used.^10^, ^11^ The ability of the ultrasound operator to optimally visualise the aortic wall interface depends on various technical factors, such as a significant difference in tissue impedance which will provide the most hyperechoic signal. This can be further obscured from the surrounding tissue in relation to the vertebral column and the ability to delineate changing interfaces.^12^ The finding that when CT and ultrasound measurements are compared in regard to accuracy, there is a difference of approximately 5 mm between modalities has been reported.^13^, ^14^

The cardiac cycle phase has also been noted as a confounding factor in the aortic measurements, with as much as a 2 mm difference between the systolic and diastolic phases.^15^ The use of electrocardiograph (ECG)‐gated ultrasound measurement for small AAA to reduce variability in measurement has been shown.^16^ The European Society of Vascular Surgery recommends that the anteroposterior (AP) measuring plane with a consistent calliper placement should be considered, a preferred method for ultrasound abdominal aortic diameter measurements.^17^ However, the guidelines also note that ‘given the variation of evidence, opinion and established routines, and the importance of training, it is not possible to specify the preferred method at this stage’.

While the ‘conventional’ anteroposterior walls in both transverse and sagittal planes may be suitable for aneurysms with less complicated geometry, there is controversy regarding the suitability of this approach for complicated, particularly tortuous aneurysms.^18^

The methodological variation in performing ultrasound assessments could potentially lead to discrepancies in care and consistent standards employed by the ultrasound practitioners. The ultrasound inter‐observer measurement variability ranges from 2 to 10 mm, and it generally tends to underestimate the maximum aortic diameter compared with CT.^19^

The maximum diameter of ultrasound is reported to be 2.7 mm, which is smaller than that of CT measurements, approximately 70% of the time.^20^ CT is generally considered to be more reproducible than ultrasound. CT also provides comprehensive 3D reconstruction of the aorta, which is essential in planning for vascular repair.^8^, ^17^, ^19^, ^21^ However, patients are subjected to radiation and contrast exposure in CT.

The previously published work in the literature found that even the inexperienced users place the calliper more optimally on the abdominal aorta than by those using the traditional ultrasound approach.^22^

**Figure 1:**
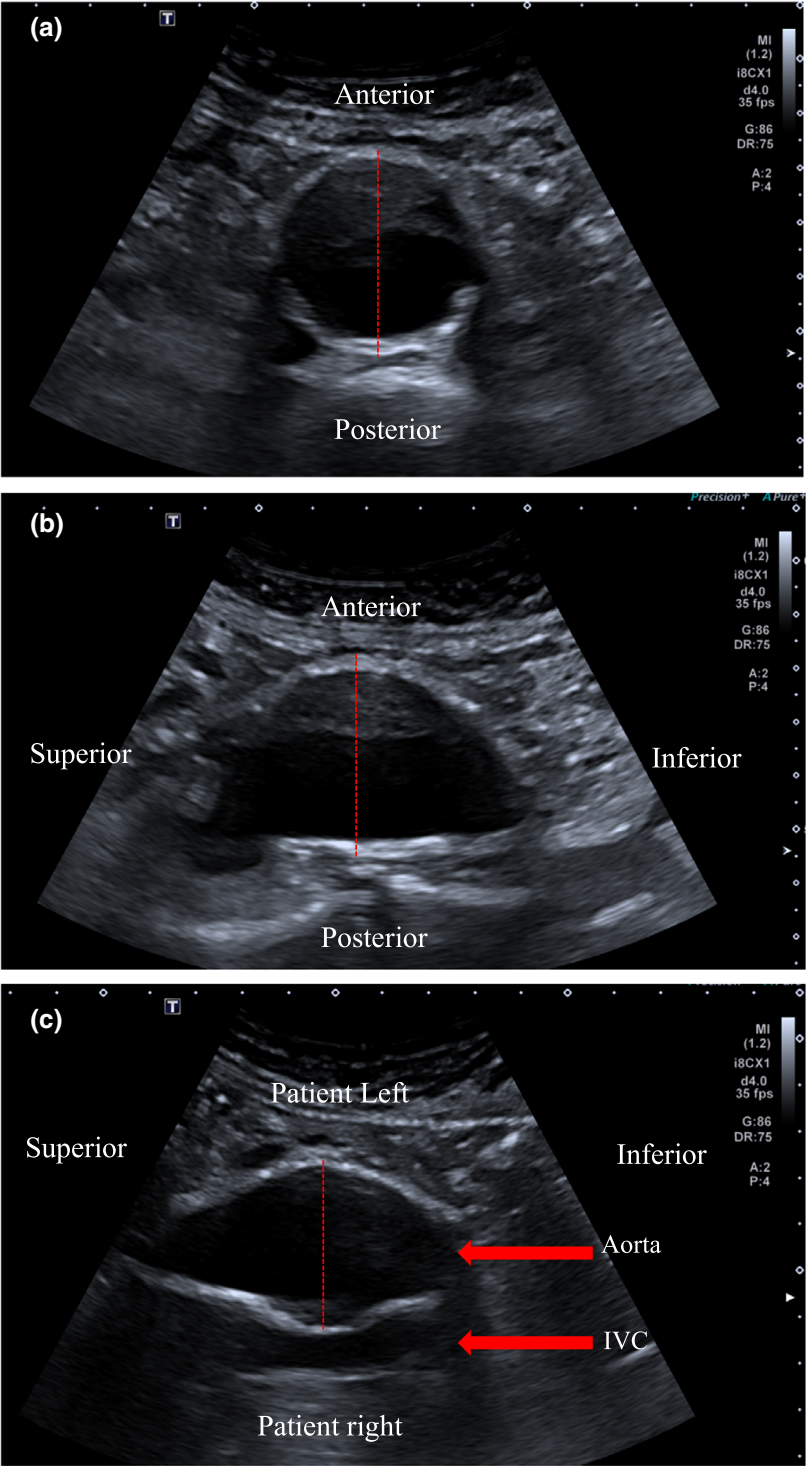
Abdominal aortic aneurysm (AAA) : (a) transverse AP diameter (TRVAPD), (b) sagittal AP diameter (SAGAPD) and (c) coronal diameter (CORRLD).

This retrospective cohort study therefore aimed to correlate three ultrasound planes: (i) transverse AP diameter (TRVAPD), (ii) sagittal AP diameter (SAGAPD) and (iii) coronal diameter (CORRLD) (Figure 1), and compared these planes with the reported maximum infrarenal abdominal aortic aneurysm on CT.

## Methods

### Audit design

**Figure 2:**
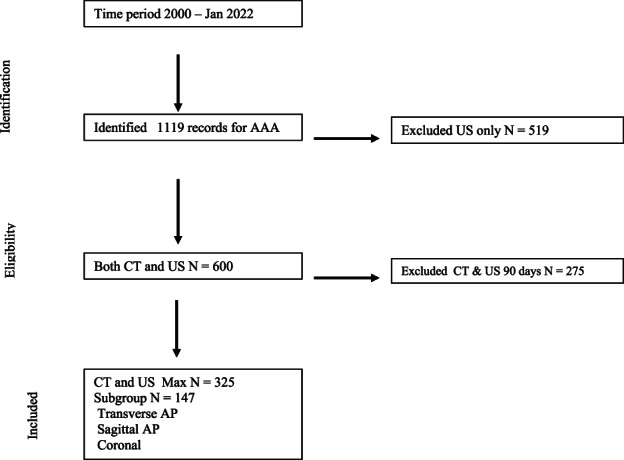
Flow chart of abdominal aortic aneurysm (AAA) population with both ultrasound and computed tomography (CT) information with less than 90 days between modalities.

A retrospective single‐centre observational study was performed, of the Otago Vascular Diagnostics database from January 2000 to January 2022. All AAA patients were identified, and those who met the threshold for intervention were included in the audit. Health Connect South (patient management system) was used to cross‐reference and identify which patients had a CT as part of their management plan, including the time difference from the ultrasound to CT (Figure 2).

A less than 90‐day difference between modalities was used as the cut‐off for inclusion in the study. Patient information collected, included: sex, age, ethnicity and other relevant clinical histories (cardiovascular disease risk factors).

The ultrasound protocol involved infrarenal abdominal aortic measurements from the anterior‐to‐posterior (AP), outer‐to‐outer wall in both transverse and sagittal planes. A right lateral decubitus viewing from the patients left was to obtain the coronal measurement of the aorta, a measurement often performed by others as the transverse measurement.^3^, ^9^ No measurements were taken perpendicular to the probe, that is, transverse in the supine position, as the level of resolution was often considered inadequate. All the ultrasound measurements were taken during the maximum systolic phase of the cardiac cycle.^15^, ^16^

The standardised Otago Vascular Diagnostics clinical assessment protocol was performed on all patients in this audit, and no additional measurements for research purposes were taken. The ultrasound and CT findings were collected separately. The ultrasound measurements were exported directly from the research database, where they were electronically embedded into the database at the time of the clinical ultrasound surveillance. This is achieved by direct communication of the XML files from the ultrasound machine to the data repository site (vascular laboratory database). The raw ultrasound data measurements were recorded in millimeters.

The CT measurement that was initially reported was used with no remeasurement of the CT performed. This information was collected by an independent member of the research team. They were given access to the patients unique identifier and date of the ultrasound scan, to aid in the collection of the CT data. This information was necessary to ensure both ultrasound and CT were performed within 90 days. The plane in which the CT measurement was taken during reporting was not recorded, so this information was unavailable. Therefore, CT reported maximum was compared against the ultrasound max irrespective of the imaging plane and then against the transverse, sagittal and coronal ultrasound planes.

Continuous data are expressed as mean (±1 standard deviation) except smoking pack‐years (non‐Gaussian distribution), which are presented as median (interquartile range). The levels of agreement between ultrasound measures (transverse AP, sagittal AP and coronal) and CT measures were assessed by calculating the bias (mean difference and standard deviation of the differences) and the 95% confidence intervals for the bias using the Bland–Altman method.^24^ Statistical analysis was performed with the StatView® version (SAS Institute, Cary, North Carolina, USA).

### Equipment

Throughout the audit, the following ultrasound platforms were used: Interest (Antares) (Siemens Medical Solutions, Inc. Mountain View, California USA), ATL 5000 (Phillips Healthcare, Corporate Centre Dr, Franklin, TN, USA) and Aplio 500 (Canon Medical Systems Coporation, Otawara, Tochigi, Japan), with the transducer frequency of CH 4–1, C5‐2 and 8C1, respectively. All B‐mode images were optimised throughout the examination. A team of four experienced vascular sonographers performed the ultrasound examinations.

### Ethics and analysis

Ethical approval was granted by the University of Otago Ethics Committee (ref.:HD22/024). This study was completed following the Helsinki declaration.

## Results

A total of 325 patients (238 male and 87 female), combined mean age 76.4 ± 7.5 years, were reviewed in this audit and found suitable for inclusion in this study with the demographics shown in Table 1. There were no significant associations observed between (CT or US) aortic size and hypertension, smoking history (current, ex‐smoker vs. never smoked) or cardiovascular disease events in this aneurysm surveillance cohort.

**Table 1:** Cohort demographics.

Variable	Female (n = 87)	Male (n = 238)
Age (years),	77.3 ± 7.7	76.1 ± 7.4
Ischaemic heart disease, n (%)	23 (26.7)	95 (40.1)
Hypertension, n (%)	65 (74.7)	157 (66.8)
Hypercholesterolaemia, n (%)	58 (66.7)	146 (61.9)
Diabetes, n (%)	11 (12.9)	26 (11.3)
History of claudication, n (%)	17 (19.5)	44 (19.2)
Obesity (noted at scan), n (%)	16 (18.8)	51 (23.4)
Body mass index (kg/m^2^)	25.7 ± 5.2	27.2 ± 5.5
Current smoker, n (%)	21 (24.1)	41 (17.2)
Ex‐smoker, n (%)	46 (52.9)	162 (68.1)
Smoking pack‐years, years	17.5 (0–30)	25.5 (10–45)
CT aortic diameter, mm	51.9 ± 12.1	57.2 ± 13.1
US max aortic diameter, mm	50.6 ± 11.7	55.7 ± 12.8
US coronal aortic diameter, mm	50.5 ± 11.7	55.2 ± 13.1
US AP TS aortic diameter, mm (n = 44/115)	48.0 ± 11.6	49.6 ± 9.4
US sagittal aortic diameter, mm (n = 42/112)	46.7 ± 9.8	49.6 ± 9.9

Continuous results are expressed as mean ± one standard deviation, except smoking pack‐years, which is expressed as median and interquartile range.

Neither body mass index (BMI), nor the sonographer's impression of patient habitus were found to be associated factors in measuring the infrarenal aorta in each of the different ultrasound planes.

The mean ultrasound anterior‐to‐posterior, outer‐to‐outer wall in both transverse and sagittal planes were 48.7 ± 10.5 mm and 48.9 ± 9.9 mm, respectively. Coronal diameter measurement of the aorta was 53.9 ± 12.8 mm in the right lateral decubitus. The mean ultrasound max was 54.3 ± 12.6 mm. The mean CT diameter measurement was 55.6 ± 12.7 mm.

**Figure 3:**
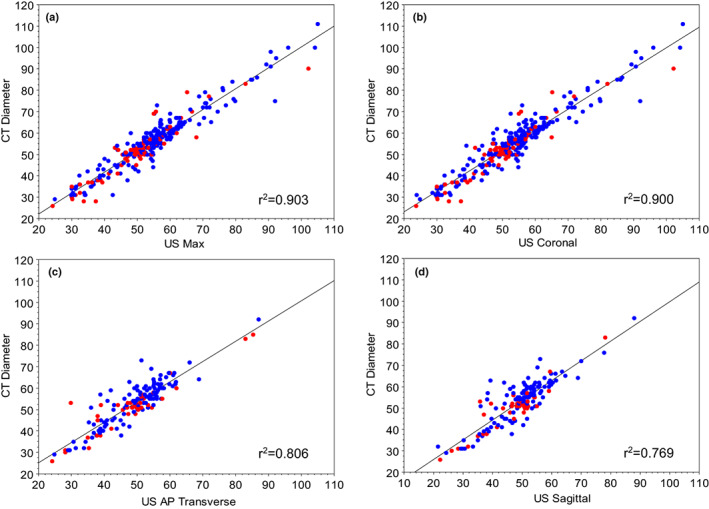
Correlations between maximum computed tomography (CT) diameter and ultrasound diameter: (a) maximum, (b) coronal, (c) transverse AP and (d) sagittal AP, split by sex (blue symbols are male and red symbols are female)

The maximum ultrasound diameter, irrespective of the plane, was then compared against the reported maximum CT diameter of the infrarenal aorta. The strongest correlation between CT max and ultrasound max was the coronal and US max measurements (r^2^ = 0.90). The AP transverse and sagittal measurements were less strongly correlated with computed tomography (CT), r^2^ = 0.81 and r^2^ = 0.80, respectively (Figure 3). In a subset of participants with all three ultrasound imaging planes (n = 147), the strongest correlation with CT diameter was with ultrasound coronal (r^2^ = 0.87), followed by ultrasound AP transverse (r^2^ = 0.81) and then US AP sagittal (r^2^ = 0.77).

**Figure 4:**
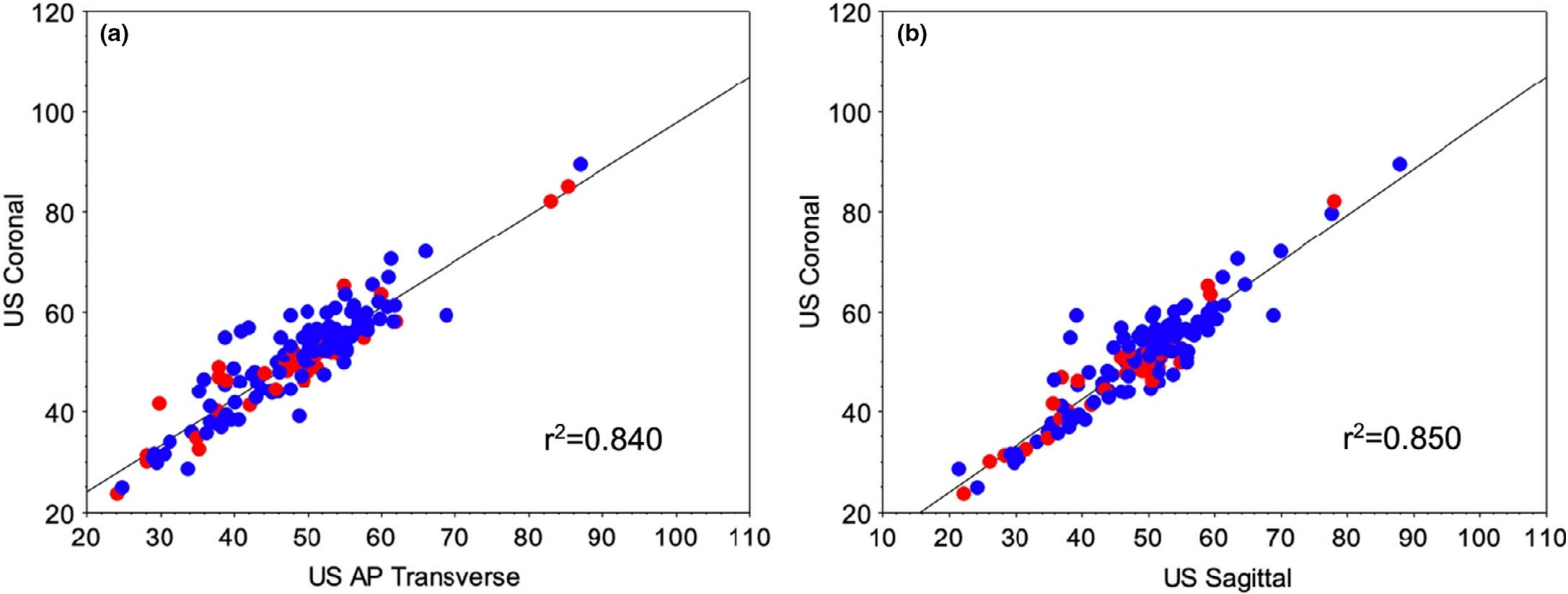
Correlation between ultrasound planes, coronal vs. (a) transverse AP and (b) sagittal AP, split by sex (blue symbols are male and red symbols are female).

When each of the ultrasound planes was compared, the strength of the correlation between coronal vs. AP transverse (r^2^ = 0.84) and AP sagittal (r^2^ = 0.85) was lesser (Figure 4).

The Bland–Altman systematic difference analysis (estimated measurement bias and 95% confidence intervals) for coronal ultrasound compared with CT diameter indicated an inter‐modality bias of 1.79 mm (95%CI 1.34–2.24, n = 325 comparisons), which was lower than for AP transverse (3.57 mm; 95%CI 2.84–4.30, n = 159 comparisons) or AP sagittal (3.75 mm; 95%CI 2.94–4.56, n = 153 comparisons) plane comparisons with CT. The estimated bias for coronal ultrasound compared with CT diameter remained similarly low (1.91 mm; 95%CI 1.26–2.56) when only those participants with all three ultrasound imaging planes (n = 147 comparisons) were included. Of note, the confidence intervals for coronal ultrasound did not overlap with the other planes, suggesting that it had a significantly (P < 0.05) better agreement with the ‘gold standard’ CT measures.

## Discussion

Ultrasound is the first modality of choice to identify the presence of AAA and has been well‐reviewed and successfully applied in clinical practice. However, there remains some debate on the optimal placement of the callipers and the optimal plane from which to obtain the measurements.^23^ As sonographers, can an improved AAA measurement using an alternative ultrasound plane be possible?

Regarding the calliper placement, the optimal posterior wall of both the transverse AP and sagittal AP aortic planes has been discussed as a difficult interface to determine. When performing an ultrasound examination, the ability to get an optimal reflective interface aids in the resolution of findings. However, too much reflection can create overly bright echoes that make placing the callipers difficult. Ultrasound physics, concerning the most effective reflective interface, ‘is expressed as R = (Z1−Z2)/(Z1 + Z2), where R is the reflection coefficient, and Z is the tissue impedance’.^10^ The bigger the difference in tissue impedance, the greater the reflection. Consequently, the ability to differentiate the posterior wall of the aorta in both the transverse AP and sagittal AP planes can be more difficult to optimise due to the significant difference in tissue impedance between the aortic wall and the tissue posterior to it (e.g. close proximity to vertebral processes), creating a strong reflective interface. This is evident when we compare the transverse AP and the sagittal AP against both the coronal and CT maximum diameters. This effect is reduced or minimised in a coronal plane as impedance mismatch in the tissue lateral to the aorta is lesser.

There was a strong level of correlation between the US coronal and CT maximum diameters in this study. Moreover, the difference in absolute values was also consistently 2 mm higher in CT measures than the coronal US plane, a value that is within that previously described in the literature.^19^ In addition to this, the coronal plane, utilising the decubitus approach, offers the sonographer the ability to more fully appreciate the contour of the aorta vessel and correct for the presence of tortuosity.

The patient's habitus and bowel gas are often reported as limiting factors in viewing the abdominal aorta. Within this study, no apparent associations were observed when investigating BMI and CT‐ultraound agreement; this is consistent with the findings of Jeeji *et al*.,^25^ within a point‐of‐care ultrasound setting, where it was concluded ‘BMI may have had some impact on image quality, it was not significant compared to those with a normal BMI’. Therefore, BMI may not be the best predictor of success when performing abdominal ultrasounds. As part of the OVD data collection, the sonographers perceived patient's body habitus as a factor that might limit optimal ultrasound imaging was recorded. When this was factored into the analysis in the differing ultrasound planes, no association was found in measuring the abdominal aorta. The waist circumference and abdominal compliance (soft abdomen vs. a more rotund girth) might better reflect limitations when performing AAA measurements, if any do exist. This may be an area for further study.

The consensus amongst the four sonographers involved in this study was that the abdominal aorta was more easily viewed (decubitus window) in the coronal plane. Of note, it is our experience that patients also appear to favour this approach as being more comfortable than any other imaging planes.

Failure to perform any transverse lateral‐ to‐lateral wall aortic measurement compared with the coronal measurement is limitation of this study. This may have offered more evidence to support an alternative measurement to better inform the ultrasound community in AAA measurements.

## Conclusion

In this single‐centre cohort study, we offer evidence that the coronal measurement is a potential preferential method for AAA surveillance. We found that AAA diameter measurements were strongly correlated with the ‘gold standard’ CT diameter measurements when obtained perpendicular to the orientation of the ultrasound transducer (coronal view). Given the apparent high degree of accuracy of this ultrasound approach, we suggest that its use may aid in the reduction in early referral for CT, making timely and more relevant referrals to help reduce the burden on an already‐stretched radiology service.

## Authorship statement

The authors acknowledge that (i) the authorship listing conforms with the journal's authorship policy and that (ii) all authors are in agreement with the content of the submitted manuscript.

## Funding

No funding information is provided.

## Conflict of Interest

The authors have no disclosures to declare or conflicts of interest.
